# Prevalence and incidence of primary lymphocytic cicatricial alopecias in the United States from 2019 to 2024

**DOI:** 10.1016/j.jdin.2026.05.004

**Published:** 2026-05-12

**Authors:** Alice Tang, Lillian Mo, Ross O’Hagan, Jordan Talia, Benjamin Ungar

**Affiliations:** Department of Dermatology, Icahn School of Medicine at Mount Sinai, New York, New York

**Keywords:** central centrifugal cicatricial alopecia, cicatricial alopecia, epidemiology, frontal fibrosing alopecia, incidence, lichen planopilaris, prevalence, scarring alopecia

*To the Editor:* Primary lymphocytic cicatricial alopecias (PLCAs), including central centrifugal cicatricial alopecia (CCCA), frontal fibrosing alopecia (FFA), and lichen planopilaris (LPP), are chronic inflammatory scalp disorders causing permanent follicular destruction, making timely diagnosis critical for hair preservation. Despite their irreversible nature and substantial psychosocial burden, US prevalence data remain limited, as subtype-specific ICD-10 codes were only introduced in October 2024.^1^

Using Epic Cosmos, a federated electronic health record network of over 300 million patients,^2^ we estimated national PLCA incidence and prevalence among all US adults with at least 2 healthcare encounters between 2019 and 2024. PLCA cases were identified via ≥2 SNOMED CT codes, which offer greater diagnostic granularity than ICD-10 alone. Annual incidence rates were calculated, and prevalence rates were stratified by sex, race, age, urban-rural status, and US census region. Given low absolute rates, 95% CIs were calculated using Wilson binomial (prevalence) and Poisson exact (incidence) approaches. This study was IRB-exempt per 45 CFR §46.102.

Six-year prevalence for any PLCA was 37.1 per 100,000, with LPP most common (24.0 per 100,000), followed by CCCA (10.0 per 100,000) and FFA (9.8 per 100,000) (Table I). Annual incidence remained stable at 8.2-9.3 per 100,000 person-years, with a slight 2020 dip likely reflecting pandemic-related diagnostic delays (Fig 1). All subtypes showed strong female predominance (57.3 vs 12.5 per 100,000). CCCA demonstrated a striking 79:1 African American-to-White prevalence ratio; among African American women aged 30-60, prevalence reached 0.15%. PLCAs were twice as prevalent in metropolitan versus rural areas (40.3 vs 20.1 per 100,000). Prevalence was highest in the Northeast across all subtypes. CCCA prevalence peaked at ages 41-50, while LPP and FFA peaked at 61-70.

**Table I:** Prevalence of all scarring alopecias by demographic characteristics

United States, 2019-2024								
Characteristic	all PLCAs		LPP		FFA		CCCA	
	*N* (%)	Prevalence per 100,000 (95% CI)	*N* (%)	Prevalence per 100,000	*N* (%)	Prevalence per 100,000	*N* (%)	Prevalence per 100,000
Overall	58,541	37.08 (36.78-37.38)	37,940	24.03 (23.79-24.27)	15,427	9.77 (9.62-9.93)	15,780	9.99 (9.84-10.15)
Sex								
Male	8246 (14.2)	11.87 (11.62-12.13)	4043 (10.7%)	5.82 (5.64-6.0)	404 (2.6%)	0.58 (0.53-0.64)	275 (1.8%)	0.4 (0.35-0.45)
Female	49,797 (85.8)	57.33 (56.83-57.84)	33,579 (89.3%)	38.66 (38.25-39.08)	14,911 (97.4%)	17.17 (16.89-17.45)	15,373 (98.2%)	17.7 (17.42-17.98)
Race/Ethnicity								
White	24,514 (43.7)	22.86 (22.57-23.14)	30,126 (83.9%)	28.09 (27.77-28.41)	12,325 (84.3%)	11.49 (11.29-11.7)	901 (5.8%)	0.84 (0.79-0.9)
Black	29,509 (52.7)	135.58 (134.04-137.14)	4181 (11.6%)	19.21 (18.64-19.8)	1724 (11.8%)	7.92 (7.56-8.3)	14,488 (93.0%)	66.57 (65.49-67.66)
Asian	1243 (2.2)	17.33 (16.39-18.32)	1109 (3.1%)	15.46 (14.58-16.4)	376 (2.6%)	5.24 (4.74-5.8)	62 (0.4%)	0.86 (0.67-1.11)
American Indian/Alaska Native	543 (1.0)	30.42 (27.97-33.09)	345 (1.0%)	19.33 (17.39-21.48)	134 (0.9%)	7.51 (6.34-8.89)	106 (0.7%)	5.94 (4.91-7.18)
Native Hawaiian/Pacific Islander	224 (0.4)	24.92 (21.86-28.40)	133 (0.4%)	14.8 (12.49-17.53)	54 (0.4%)	6.01 (4.6-7.84)	20 (0.1%)	2.22 (1.44-3.44)
Age group (y)								
0-10	3436 (3.0)	9.93 (9.60-10.27)	979 (1.3%)	2.83 (2.66-3.01)	143 (0.4%)	0.41 (0.35-0.49)	691 (2.2%)	2 (1.85-2.15)
11-17	7022 (6.2)	22.15 (21.64-22.68)	2693 (3.5%)	8.5 (8.18-8.82)	628 (2.0%)	1.98 (1.83-2.14)	2352 (7.6%)	7.42 (7.13-7.73)
18-30	10,458 (9.2)	15.77 (15.47-16.08)	3672 (4.7%)	5.54 (5.36-5.72)	771 (2.4%)	1.16 (1.08-1.25)	3043 (9.8%)	4.59 (4.43-4.76)
31-40	9834 (8.6)	33.12 (32.47-33.78)	4480 (5.8%)	15.09 (14.65-15.54)	1486 (4.6%)	5.0 (4.76-5.27)	3523 (11.4%)	11.87 (11.48-12.26)
41-50	19,094 (16.8)	43.22 (42.62-43.84)	12,079 (15.6%)	27.34 (26.86-27.84)	5215 (16.2%)	11.81 (11.49-12.13)	6008 (19.4%)	13.6 (13.26-13.95)
51-60	28,928 (25.4)	39.16 (38.71-39.62)	16,559 (21.4%)	22.42 (22.08-22.76)	6701 (20.8%)	9.07 (8.86-9.29)	9531 (30.8%)	12.9 (12.65-13.16)
61-70	12,293 (10.8)	42.92 (42.17-43.69)	12,134 (15.7%)	42.37 (41.62-43.13)	5733 (17.8%)	20.02 (19.51-20.54)	2322 (7.5%)	8.11 (7.78-8.44)
71-80	4891 (4.3)	30.19 (29.36-31.05)	5948 (7.7%)	36.72 (35.8-37.66)	2693 (8.4%)	16.62 (16.01-17.26)	564 (1.8%)	3.48 (3.21-3.78)
81-90	17,184 (15.1)	38.32 (37.76-38.90)	18,082 (23.3%)	40.33 (39.74-40.92)	8426 (26.2%)	18.79 (18.39-19.2)	2886 (9.3%)	6.44 (6.21-6.68)
91+	679 (0.6)	11.18 (10.37-12.05)	869 (1.1%)	14.31 (13.39-15.29)	373 (1.2%)	6.14 (5.55-6.8)	75 (0.2%)	1.24 (0.99-1.55)
US census region								
Northeast	14,169 (24.3)	46.44 (45.68-47.21)	9203 (24.3%)	30.16 (29.55-30.79)	3999 (26.0%)	13.11 (12.71-13.52)	4555 (29.1%)	14.93 (14.5-15.37)
Midwest	14,970 (25.7)	39.23 (38.60-39.86)	10,865 (28.7%)	28.47 (27.94-29.01)	4004 (26.0%)	10.49 (10.17-10.82)	3563 (22.8%)	9.34 (9.03-9.65)
South	22,385 (38.5)	37.09 (36.60-37.57)	11,579 (30.6%)	19.18 (18.84-19.54)	5041 (32.8%)	8.35 (8.12-8.59)	7038 (45.0%)	11.66 (11.39-11.94)
West	6671 (11.5)	23.61 (23.05-24.18)	6217 (16.4%)	22.0 (21.46-22.56)	2345 (15.2%)	8.3 (7.97-8.64)	480 (3.1%)	1.7 (1.55-1.86)
Rural/Urban status								
Metropolitan (RUCA 1-3)	53,863 (92.1)	40.25 (39.91-40.59)	33,074 (87.3%)	24.71 (24.45-24.98)	13,650 (88.6%)	10.2 (10.03-10.37)	15,033 (95.3%)	11.23 (11.06-11.41)
Rural (RUCA 4-10)	4614 (7.9)	20.11 (19.53-20.69)	4812 (12.7%)	20.97 (20.38-21.57)	1759 (11.4%)	7.67 (7.32-8.03)	736 (4.7%)	3.21 (2.98-3.45)

*CCCA*, Central centrifugal cicatricial alopecia; *FFA*, frontal fibrosing alopecia; *LPP*, lichen planopilaris; *PLCA*, primary lymphocytic cicatricial alopecia; *RUCA*, Rural-Urban Commuting Area.

**Fig 1:**
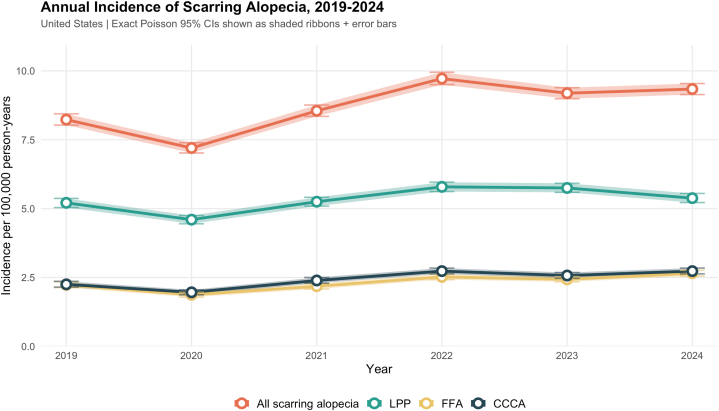
Annual incidence of scarring alopecias in the United States, 2019-2024. Incidence is reported per 100,000 person-years. Shaded ribbons and error bars represent exact Poisson 95% confidence intervals. *CCCA*, Central centrifugal cicatricial alopecia; *FFA*, frontal fibrosing alopecia; *LPP*, lichen planopilaris; *PLCA*, primary lymphocytic cicatricial alopecia.

This is the first nationwide prevalence study of scarring alopecia subtypes. The finding of 0.15% CCCA prevalence among African American women aged 30-60 suggests that scarring alopecias may be more common than appreciated in specific populations. Our estimates closely align with previous NYC-based studies,^3^^,^^4^ though slightly lower rates likely reflect our nationwide scope, given that urban and Northeast populations demonstrated higher prevalence compared to national averages.

This study has limitations inherent to large-scale EMR analyses. Misdiagnosis is a concern when using diagnosis code–based strategies, as scarring hair loss diagnosis often requires trichoscopy and/or biopsy by specialists. In our study, 89.1% of all diagnoses were made by dermatologists (87.2% of LPP, 90.7% of FFA, and 95.1% of CCCA), providing some reassurance regarding diagnostic accuracy. To assess external validity, we evaluated SNOMED-based alopecia areata prevalence rates within Epic Cosmos (0.1886%) and found they closely match previously published rates.^5^ Because patients may carry multiple diagnoses, summed subtype prevalences exceed total PLCA prevalence. True PLCA prevalence is likely underestimated in underserved population, and higher rates in metropolitan areas and the Northeast groups may reflect greater access to dermatologic care rather than true variation in disease burden. Nevertheless, these findings establish a national epidemiologic baseline for PLCAs and underscore the need for equitable access to timely diagnosis which can impact prognosis..

## Conflicts of interest

Dr Talia has served as a consultant for Abbvie, Arcutis Biotherapeutics, Bristol-Meyers Squibb, Calliditas Therapeutics, Galderma, Johnson & Johnson, Leo Pharma, Novartis, Navigator Medicines, Primus Pharmaceuticals, Sanofi Genzyme, Stifel Financial, and UCB. He serves or has served as an investigator for Attovia Therapeutics, LEO Pharma, Priovant Therapeutics, and Sanofi. Dr Ungar is an employee of Mount Sinai and has received research funds (grants paid to the institution) from: Bristol Myers Squibb, Incyte, Rapt Therapeutics, Pfizer, and Sanofi. He is also a consultant for AbbVie, Arcutis Biotherapeutics, Apogee Therapeutics, Bristol Myers Squibb, Botanix Pharmaceuticals, Castle Biosciences, Ebla Holdco, Fresenius Kabi, Galderma, J&J, Leo Pharma, Lilly, Nektar Therapeutics, Pfizer, Primus Pharmaceuticals, Sanofi, Sun Pharma, UCB, Veradermics, VRG Therapeutics. Authors Tang, Mo, and Dr O’Hagan have no conflicts of interest to declare.
